# The role of megakaryocytes in breast cancer metastasis to bone

**DOI:** 10.1186/1750-9378-6-S1-A7

**Published:** 2011-08-11

**Authors:** Rose Mary Stiffin, Jana Miles, Andrea Mastro, Donna Sosnoski, Yu-Chi Chen

**Affiliations:** 1 Florida Memorial University, Florida, USA; 2 The Pennsylvania State University, Pennsylvania, USA

## Background

Each year, thousands of individuals are diagnosed with breast cancer. If detected early, the survival rate is close to 90%; however, that rate drops to around 4% if the breast cancer metastasizes. Breast cancer cells often and typically metastasize to long bones, such as femur and tibia. In long bones are large, multinucleated cells known as megakaryocytes, which produce platelets. Many cancer patients display an increase in platelet production; this platelet increase leads to thromboembolism, which is the leading cause of death for cancer victims. Increased platelet production may be due in part to an increase in megakaryopoiesis. The primary aim of this research is to determine the presence of mature, von Willebrand Factor-expressing megakaryocytes in mice injected with cancer cells, either intracardially or in the mammary glands. The secondary aim is to determine the presence of cancer cells (GFP+) in the bone marrow, a clear indication of metastasis.

## Methods

Athymic mice were intracardially or mammary gland injected with cancerous human MDA-MB-231 human metastatic breast cancer cells stably transfected with a marker protein (green fluorescent protein or GFP). The intracardiac injection normally leads to bone metastasis, while the mammary gland injection leads to growth of the tumor in the mammary gland with little or no metastasis. The mice were sacrificed on a time course. The long bones of the mice were fixed and sectioned for both immunohistochemical (IHC) and hematoxylin & Eosin (H&E) staining. The avidin-biotin complex IHC was carried out with an antibody to von Willebrand Factor, which is expressed by megakaryocytes. H&E allows identification of megakaryocytes based on size and morphology.

## Results

Femurs from flank injected mice sacrificed 3 – 4 weeks post injection showed no evidence of MDA-MB-231 breast cancer cells. At two weeks post injection versus three weeks post injection, VWF^+^ MKs in tibia from mice intracardially injected with PBS (control) and MDA-MB-231 cells were significantly lower than MDA-MB-231-BRMS cells. Given that BRMS-1 is a transcription factor and metastasis suppressor, it is possible that over time it influences the down-regulation of megakaryopoiesis.

## Acknowledgements

Publication of this article was funded in part by the University of Florida Open-Access Publishing Fund.

